# Supplementary material for: Paternal over- and under-nutrition programme fetal and placental development in a sex-specific manner in mice
Source: eLife. 2026 Jul 20;15:RP109392. doi: 10.7554/eLife.109392 (PMC13384496; doi:10.7554/eLife.109392)
Supplement: Supplementary file 2. [file elife-109392-supp2.docx]

**Supplementary file 2.** Sexing PCR primer sequences.

| **Gene loci name** | **Primer Sequences** | | **Amplicon Length (bp)** |
| --- | --- | --- | --- |
|  | **Forward Primer** | **Reverse Primer** |  |
| Sry | gtgagaggcacaagttggc | ctctgtgtaggatcttcaatc | 147 |
| Dxnds3 | gagtgcctcatctatacttacag | tctagttcattgttgattagttgc | 244 |
