# Supplementary material for: Paternal over- and under-nutrition programme fetal and placental development in a sex-specific manner in mice
Source: eLife. 2026 Jul 20;15:RP109392. doi: 10.7554/eLife.109392 (PMC13384496; doi:10.7554/eLife.109392)
Supplement: Peer review history

# Editor report: eLife Assessment

**DOI:** 10.7554/eLife.109392.4.sa0

**Authors:** Karen Forbes

**Affiliations:** University of Leeds United Kingdom

**Keywords:** Convincing, Important

This **important** study demonstrates that paternal diet influences not only testicular morphology but also placental and fetal development, supporting a role for paternal contributions to offspring health. The study also considers potential links between the microbiome and male reproductive health. By combining transcriptomic and histological analyses across multiple tissues, the evidence supporting the central conclusions of the study is **convincing**.

# Referee report: Reviewer #1 (Public review)

**DOI:** 10.7554/eLife.109392.4.sa1

[Editors' note: this version has been assessed by the Reviewing Editor without further input from the original reviewers. The authors have addressed the comments raised in the previous round of review.]

Summary:

Morgan et al. studied how paternal dietary alteration influenced testicular phenotype, placental and fetal growth using a mouse model of paternal low protein diet (LPD) or Western Diet (WD) feeding, with or without supplementation of methyl-donors and carriers (MD). They found diet- and sex-specific effects of paternal diet alteration. All experimental diets decreased paternal body weight and the number of spermatogonial stem cells, while fertility was unaffected. WD males (irrespective of MD) showed signs of adiposity and metabolic dysfunction, abnormal seminiferous tubules and dysregulation of testicular genes related to chromatin homeostasis. Conversely, LPD induced abnormalities in the early placental cone, fetal growth restriction and placental insufficiency, which was partly ameliorated by MD. The paternal diets changed placental transcriptome in a sex-specific manner and led to a loss of sexual dimorphism in the placental transcriptome. These data provide a novel insight on how paternal health can affect the outcome of pregnancies, which is often overlooked in prenatal care.

Strengths:

The authors have performed a well-designed study using commonly used mouse models of paternal underfeeding (low protein) and overfeeding (Western diet). They performed comprehensive phenotyping at multiple timepoints including of the fathers, the early placenta and late gestation feto-placental unit. The inclusion of both testicular and placental morphological and transcriptomic analysis is a powerful non-biased tool for such exploratory observational studies. The authors describe changes in testicular gene expression revolving around histone (methylation) pathways that are linked to altered offspring development (H3.3 and H3K4), which is in line with hypothesised paternal contributions to offspring health. The authors report sex differences in control placentas that mimic those in humans, providing potential for translatability of the findings. The exploration of sexual dimorphism (often overlooked) and its absence in response to dietary modification is novel and contributes to the evidence-base for the inclusion of both sexes in developmental studies.

Comments on revised version:

The authors have done a great job addressing my concerns. The description of the data analysis and the figures are now much clearer. The inclusion of the potential links between the microbiome and male reproductive fitness is informative and improves the flow of the discussion.

# Referee report: Reviewer #2 (Public review)

**DOI:** 10.7554/eLife.109392.4.sa2

Summary:

The authors investigated the effects of a low-protein diet (LPD) and a high sugar- and fat-rich diet (Western diet, WD) on paternal metabolic and reproductive parameters and feto-placental development and gene expression. They did not observe significant effects on fertility; however, they reported gut microbiota dysbiosis, alterations in testicular morphology, and severe detrimental effects on spermatogenesis. In addition, they examined whether the adverse effects of these diets could be prevented by supplementation with methyl donors. Although LPD and WD showed limited negative effects on paternal reproductive health (with no impairment of reproductive success), the consequences on fetal and placental development were evident and, as reported in many previous studies, were sex-dependent.

Strengths:

This study is of high quality and addresses a research question of great global relevance, particularly in light of the growing concern regarding the exponential increase in metabolic disorders, such as obesity and diabetes, worldwide. The work highlights the importance of a balanced paternal diet in regulating the expression of metabolic genes in the offspring at both fetal and placental levels. The identification of genes involved in metabolic pathways that may influence offspring health after birth is highly valuable, strengthening the manuscript and emphasizing the need to further investigate long-term outcomes in adult offspring.

The histological analyses performed on paternal testes clearly demonstrate diet-induced damage. Moreover, although placental morphometric analyses and detailed histological assessments of the different placental zones did not reveal significant differences between groups, their inclusion is important. These results indicate that even in the absence of overt placental phenotypic changes, placental function may still be altered, with potential consequences for fetal programming.

Comments on revised version:

The authors have adequately addressed all my previous comments.

# Author comment: Author response

**DOI:** 10.7554/eLife.109392.4.sa3

**Authors:** Hannah L Morgan, Nader Eid, Nadine Holmes, Matthew Carlile, Sonal Henson, Fei Sang, Victoria Wright, Marcos Castellanos-Uribe, Iqbal Khan, Nazia Nazar, Sean May, Rod Mitchell, Federica Lopes, Robert S Robinson, A Augusto Coppi, Vipul Batra, Adam J Watkins

**Affiliations:** University of Manchester Manchester United Kingdom; University of Nottingham Nottingham United Kingdom; University of Nottingham Nottingham United Kingdom; University of Nottingham Nottingham United Kingdom; University of Nottingham Nottingham United Kingdom; University of Nottingham Nottingham United Kingdom; University of Nottingham Nottingham United Kingdom; University of Nottingham Nottingham United Kingdom; University of Nottingham Nottingham United Kingdom; University of Nottingham Nottingham United Kingdom; University of Nottingham Loughborough United Kingdom; University of Edinburgh Edinburgh United Kingdom; University of Dundee Dundee United Kingdom; University of Nottingham Nottingham United Kingdom; University of Bristol Bristol United Kingdom; University of Sheffield Sheffield United Kingdom; University of Sheffield Sheffield United Kingdom

The following is the authors’ response to the previous reviews

**Public Reviews:**

**Reviewer #1 (Public review):**

Summary:

Morgan et al. studied how paternal dietary alteration influenced testicular phenotype, placental and fetal growth using a mouse model of paternal low protein diet (LPD) or Western Diet (WD) feeding, with or without supplementation of methyl-donors and carriers (MD). They found diet- and sex-specific effects of paternal diet alteration. All experimental diets decreased paternal body weight and the number of spermatogonial stem cells, while fertility was unaffected. WD males (irrespective of MD) showed signs of adiposity and metabolic dysfunction, abnormal seminiferous tubules and dysregulation of testicular genes related to chromatin homeostasis. Conversely, LPD induced abnormalities in the early placental cone, fetal growth restriction and placental insufficiency, which was partly ameliorated by MD. The paternal diets changed placental transcriptome in a sex-specific manner and led to a loss of sexual dimorphism in the placental transcriptome. These data provide a novel insight on how paternal health can affect the outcome of pregnancies, which is often overlooked in prenatal care.

Strengths:

The authors have performed a well-designed study using commonly used mouse models of paternal underfeeding (low protein) and overfeeding (Western diet). They performed comprehensive phenotyping at multiple timepoints including of the fathers, the early placenta and late gestation feto-placental unit. The inclusion of both testicular and placental morphological and transcriptomic analysis is a powerful non-biased tool for such exploratory observational studies. The authors describe changes in testicular gene expression revolving around histone (methylation) pathways that are linked to altered offspring development (H3.3 and H3K4), which is in line with hypothesised paternal contributions to offspring health. The authors report sex differences in control placentas that mimic those in humans, providing potential for translatability of the findings. The exploration of sexual dimorphism (often overlooked) and its absence in response to dietary modification is novel and contributes to the evidence-base for the inclusion of both sexes in developmental studies.

Comments on revised version:

The authors have done a great job addressing my concerns. The description of the data analysis and the figures are now much clearer. The inclusion of the potential links between the microbiome and male reproductive fitness is informative and improves the flow of the discussion.

**Reviewer #2 (Public review):**

Summary:

The authors investigated the effects of a low-protein diet (LPD) and a high sugar- and fat-rich diet (Western diet, WD) on paternal metabolic and reproductive parameters and feto-placental development and gene expression. They did not observe significant effects on fertility; however, they reported gut microbiota dysbiosis, alterations in testicular morphology, and severe detrimental effects on spermatogenesis. In addition, they examined whether the adverse effects of these diets could be prevented by supplementation with methyl donors. Although LPD and WD showed limited negative effects on paternal reproductive health (with no impairment of reproductive success), the consequences on fetal and placental development were evident and, as reported in many previous studies, were sex-dependent.

Strengths:

This study is of high quality and addresses a research question of great global relevance, particularly in light of the growing concern regarding the exponential increase in metabolic disorders, such as obesity and diabetes, worldwide. The work highlights the importance of a balanced paternal diet in regulating the expression of metabolic genes in the offspring at both fetal and placental levels. The identification of genes involved in metabolic pathways that may influence offspring health after birth is highly valuable, strengthening the manuscript and emphasizing the need to further investigate long-term outcomes in adult offspring.

The histological analyses performed on paternal testes clearly demonstrate diet-induced damage. Moreover, although placental morphometric analyses and detailed histological assessments of the different placental zones did not reveal significant differences between groups, their inclusion is important. These results indicate that even in the absence of overt placental phenotypic changes, placental function may still be altered, with potential consequences for fetal programming.

Comments on revised version:

The authors have adequately addressed all my previous comments.

We would like to thank the Editor and Reviewers for their consideration and thoughtful comments.

**Recommendations for the authors:**

**Reviewer #1 (Recommendations for the authors):**

It was a little difficult seeing exactly what had changed in the manuscript without going back to the original version as not all changes were marked yellow in the revised version. In future, I would recommend clearly labelling all changes to aid the referee.

We apologise to the reviewer for the difficulty in seeing where the changes had been made. We acknowledge their comments for subsequent manuscripts and thank them for their time, consideration and comments.

Small comments:

(1) I noted the description of the statistical analysis now includes the addition of paternal age/diet duration in the generalised mixed model for the late gestation cohort. Was this also done for the early gestation cohort? If not, why not?

For the data presented in Figure 6, each data point was obtained from a separate male. As such, we were not able to factor in male effects, as no male sired more than one litter (Figure 6A). Additionally, only one conceptus per male was analysed for ECP area and development meaning paternal age effects could not be accounted for.

(2) The legend of Figure 2 states that "Data were analysed using either a one-way ANOVA with Holm-Sidak post hoc tests for multiple comparison respectively". Is some text missing here?

We thank the reviewer for spotting this typographical error. This has now been corrected and reads “Data were analysed using a one-way ANOVA with Holm-Sidak post hoc tests for multiple comparison”.

(3) Figure 1 remains low resolution in the reviewer's copy. If possible, it would be good to upload a higher resolution figure during production of the article.

We apologies that the resolution of this figure was still low for the Reviewer. We have checked the dpi and it is 300x300. However, we will ensure the quality is as high as possible during production.

**Reviewer #2 (Recommendations for the authors):**

One minor remaining issue: the caption of Figure 3 still contains the phrase "non-fasting metabolic status", which should be deleted from this sentence.

We thank the reviewer for spotting this typographical mistake. This has now been corrected.
